# Padrões de trajetórias de autoavaliação de saúde e fatores associados no ELSA-Brasil

**DOI:** 10.11606/s1518-8787.2024058005580

**Published:** 2024-12-16

**Authors:** Camila Arantes Ferreira Brecht D’Oliveira, Daniela Polessa Paula, Aline Silva-Costa, Odaleia Barbosa de Aguiar, Lidyane V. Camelo, Ana Luísa Patrão, Maria de Jesus Mendes da Fonseca, Rosane Härter Griep

**Affiliations:** I Ministério da Saúde. Secretaria de Vigilância em Saúde e Ambiente. Departamento de Análise Epidemiológica e Vigilância de Doenças Crônicas Não Transmissíveis. Brasília, DF, Brasil; II Instituto Brasileiro de Geografia e Estatística. Escola Nacional de Ciências Estatísticas. Rio de Janeiro, RJ, Brasil; III Universidade do Estado do Rio de Janeiro. Instituto de Matemática e Estatística. Rio de Janeiro, RJ, Brasil; IV Universidade Federal do Triângulo Mineiro. Instituto de Ciências da Saúde. Departamento de Saúde Coletiva. Uberaba, MG, Brasil; V Universidade do Estado do Rio de Janeiro. Instituto de Nutrição. Departamento de Nutrição Aplicada. Rio de Janeiro, RJ, Brasil; VI Universidade Federal de Minas Gerais. Faculdade de Medicina. Departamento de Medicina Preventiva e Social. Belo Horizonte, MG, Brasil; VII Universidade do Porto. Faculdade de Psicologia e de Ciências da Educação. Centro de Psicologia da Universidade do Porto. Porto, Portugal; VIII Fundação Oswaldo Cruz. Escola Nacional de Saúde Pública Sérgio Arouca. Departamento de Epidemiologia e Métodos Quantitativos. Rio de Janeiro, RJ, Brasil; IX Fundação Oswaldo Cruz. Instituto Oswaldo Cruz. Laboratório de Educação em Ambiente e Saúde. Rio de Janeiro, RJ, Brasil

**Keywords:** Trajetórias de Saúde, Autoavaliação de Saúde, Análise de Classes Latentes, Brasil

## Abstract

Descrever padrões de trajetórias de autoavaliação de saúde (AAS) e investigar sua associação com fatores sociodemográficos, ocupacionais e de saúde.

Amostra composta por 7.738 servidores públicos ativos do Estudo Longitudinal de Saúde do Adulto (ELSA-Brasil) avaliados entre 2008 e 2020. Utilizando-se a curva de crescimento de classe latente, foram definidos os padrões de trajetórias da AAS, obtidos em 11 pontos no tempo. Modelos logísticos multinomiais foram testados para analisar associações entre as exposições e os padrões de trajetórias de AAS.

Três padrões de trajetórias de AAS emergiram: i- boa, ii- regular e iii-ruim (29%, 61% e 10% dos participantes, respectivamente). Após ajustes, tiveram maiores chances de serem classificados com padrões de trajetória de AAS ruim, comparado à boa, o sexo feminino, a raça/ cor autorreferida parda, referir frequente conflito do trabalho para a família ou da família para o trabalho. Além disso, os fatores associados a maiores chances de apresentar padrão de trajetória de AAS ruim ou regular, comparados à boa, foram escolaridade até o ensino médio, menor renda, trabalho passivo, alto desgaste no trabalho, baixo apoio social, ocupação manual, percepção de escassez de tempo para o autocuidado e lazer, ter sobrepeso ou obesidade, um estilo de vida não saudável e comorbidades.

Condições socioeconômicas e ocupacionais adversas, estilo de vida não saudável e comorbidades foram associadas ao pior padrão de trajetórias de AAS.

## INTRODUÇÃO

 A autoavaliação de saúde (AAS) é um indicador baseado na perspectiva individual, amplamente utilizado na avaliação da saúde global ^1^^,^^2^ e considerado um bom preditor de mortalidade ^2^^,^^3^ . A associação da AAS com morbimortalidade está bastante difundida, sendo evidenciada concordância de 80% com a avaliação clínica da presença ou ausência de condição crônica de saúde ^1–3^ . Aliado a esses aspectos, a AAS é também considerada um indicador de fácil aplicação, precisão e baixo custo ^1^^–^^3^ . 

 Em estudos epidemiológicos, a AAS tem sido mensurada, predominantemente, em um único momento ^3^^,^^4^ . Em estudo sobre mortalidade ^3^ , a capacidade preditiva da AAS em um único momento mostrou um efeito dependente do tempo, ou seja, foi atenuada com o passar do tempo ^3^ . O conhecimento das trajetórias de AAS permite distinguir o estado de saúde das pessoas de forma consistente ou intermitente ao longo do tempo ^5^ , sendo útil para direcionar os esforços na prevenção de doenças ^6^ . 

 Estudos que analisam a AAS ao longo do tempo são mais recentes, identificando seu declínio mais acentuado entre pessoas do sexo feminino, com idade avançada e baixos níveis de escolaridade e renda ^4^^,^^6^^–^^8^ , bem como entre aqueles com um estilo de vida marcado pelo tabagismo, consumo de álcool, sedentarismo ou baixos níveis de atividade física no lazer, consumo de frutas e hortaliças ou com múltiplas condições crônicas de saúde ^4^^–^^9^ . 

 No que se refere aos fatores ocupacionais associados à AAS ao longo do tempo, a literatura internacional mostra que o trabalho repetitivo, de alta demanda psicológica, baixo apoio social ^2000^ e a insegurança relacionada ao trabalho ^2019^ estão associados aos declínios da AAS. Para trabalhadores que apresentam saúde boa antes da aposentadoria, o status ocupacional baixo (trabalhadores da manutenção, limpeza, construção entre outros), o trabalho fisicamente extenuante e a tensão no trabalho foram associados ao maior risco de declínio da AAS durante a transição para a aposentadoria ^2020^ . No Brasil, até onde foi possível buscar, apenas um artigo investigou a relação entre fatores ocupacionais e mudanças na AAS entre dois momentos, durante 10 anos de acompanhamento, utilizando o modelo de multiestado de Markov ^2020^ . Os resultados deste estudo evidenciaram que pessoas que realizam trabalho passivo (classificação que combina baixas demandas psicológicas ao baixo controle no trabalho) ou de alta exigência (classificação que combina altas demandas psicológicas ao baixo controle no trabalho) têm menos risco de transição de AAS regular para boa ^2021^ . 

O presente estudo teve como objetivo descrever padrões de trajetórias de AAS em 11 anos de seguimento, além de investigar fatores sociodemográficos, ocupacionais e de saúde associados aos padrões de trajetórias de AAS em uma coorte brasileira.

## MÉTODOS

### Desenho de Estudo e Participantes

 O Estudo Longitudinal de Saúde do Adulto (ELSA-Brasil), coorte multicêntrica composta na linha de base por 15.105 funcionários públicos ativos e aposentados, entre 35 e 74 anos, abrange seis instituições públicas de ensino superior e pesquisa do Brasil ^2021^ . A coleta de dados é realizada por meio de exames e entrevistas presenciais e por acompanhamento telefônico anual. Todas as ondas do estudo seguem procedimentos padronizados e são conduzidas por equipe devidamente treinada e certificada ^2015^^–^^2013^ . Foram incluídos, no presente estudo, os participantes que eram ativos tanto na linha de base (2008–2010), como na segunda (2012–2014) e na terceira onda do estudo (2017–2019). Estes foram entrevistados por meio do monitoramento telefônico anual (2009 – até 28/12/2020). 

**Figura 1.**
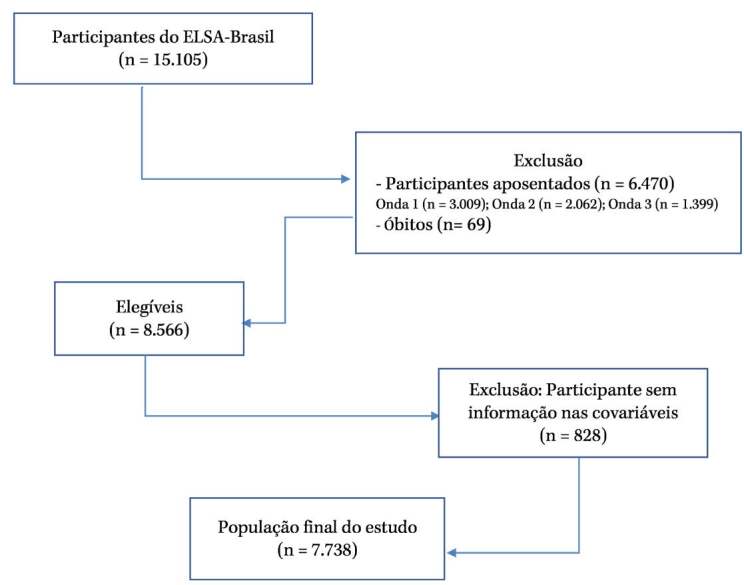
Fluxograma dos participantes incluídos nas análises. Estudo Longitudinal de Saúde do Adulto (ELSA–Brasil 2008–2020).

 Foram excluídos (i) os aposentados (n = 6.470), tanto pela ausência de informações ocupacionais deste grupo na linha de base quanto pelo fato de aqueles que se aposentaram ao longo do acompanhamento apresentarem um perfil de comportamentos de saúde distinto dos trabalhadores ativos, (ii) os óbitos (n = 69), (iii) os participantes que se declararam amarelos (n = 198) ou indígenas (n = 74), dado pequeno número de participantes em cada categoria, e (iv) aqueles que não responderam a alguma das variáveis de interesse deste estudo (n = 556). A amostra final, considerando os trabalhadores ativos em todo período de seguimento do estudo, foi composta por 7.738 participantes ( Figura 1 ). 

O estudo foi aprovado pelos comitês de pesquisa e ética das instituições envolvidas e todos os participantes assinaram o termo de consentimento livre e esclarecido.

## VARIÁVEIS DE ESTUDO

### Desfecho: Autoavaliação de saúde (AAS)

Tanto nas três ondas do estudo quanto nas ligações anuais de seguimento, foi feita a seguinte pergunta: “De um modo geral, em comparação a pessoas da sua idade, como o(a) senhor(a) considera o seu estado de saúde?”. As opções de resposta foram: “muito bom”, “bom”, “regular”, “ruim” e “muito ruim”. Dessa forma, a autoavaliação de cada participante poderia variar de 1 (muito bom) até 5 (muito ruim), em 11 pontos no tempo (três ondas e oito ligações anuais de seguimento) no período de condução do estudo (2008 a 2020). Após a aplicação do método de curva de crescimento de classe latente (LCGM), descrito detalhadamente no item análise de dados, foram identificados três padrões de trajetórias de AAS, denominados “boa”, “regular” e “ruim”.

## VARIÁVEIS DE EXPOSIÇÃO

Para o presente estudo, foram avaliadas as variáveis socioeconômicas, ocupacionais e de saúde mensuradas na linha de base do estudo.

### Variáveis Sociodemográficas

 Idade (contínua); sexo (masculino; feminino); raça/cor da pele autorreferida (branca; parda; preta); escolaridade (Até ensino médio completo; universitário; pós-graduado); renda líquida familiar *per capita* , dividida em salários com base no salário mínimo de 2008 – R$ 415,00 (≤ 3 salários mínimos; > 3 salários mínimos); situação conjugal (com companheiro(a); sem companheiro(a)). 

### Variáveis Ocupacionais

 Estresse no trabalho: mensurado por meio da versão brasileira do questionário *Swedish demand control support questionnaire* (DCSQ), abrange a demanda psicológica, o controle e o apoio social no trabalho. Neste estudo, o item sobre trabalho repetitivo foi retirado, considerando a análise da estrutura dimensional do questionário no contexto brasileiro ^2010^ . Os escores do DCSQ foram somados e dicotomizados em alto e baixo, usando a mediana das dimensões como ponto de corte (demanda = 14; controle = 17; apoio = 20). A variável estresse no trabalho foi categorizada em quadrantes: baixo desgaste no trabalho (baixa demanda psicológica/alto controle); trabalho ativo (alta demanda psicológica/alto controle); trabalho passivo (baixa demanda psicológica/baixo controle); e alto desgaste no trabalho (alta demanda psicológica/baixo controle). O apoio social foi categorizado como alto e baixo, a partir da mediana da distribuição dos escores. 

Carga horária de trabalho: classificada em ≤ 40 horas/semana e > 40 horas/semana.

Natureza da Ocupação: variável obtida por meio da seguinte pergunta: “Por favor, descreva as principais atividades que o(a) senhor(a) desenvolve no seu dia a dia de trabalho na (nome da instituição)”. A classificação das ocupações, de acordo com a natureza das tarefas necessárias para sua realização, considerou as habilidades adequadas para a realização de tarefas manuais (ou não) e rotineiras (ou não), em quatro categorias: não manual não rotineiro, não manual rotineiro, manual não rotineiro, manual rotineiro. Neste estudo, as categorias manual não rotineiro e manual rotineiro foram agrupadas em “manual”.

 Conflito trabalho-família. O conflito trabalho-família foi mensurado por quatro perguntas ^2016^ . A primeira aborda o conflito do trabalho para a família (tempo) – “Demandas (exigências ou solicitações) do trabalho o/a impedem de passar a quantidade de tempo desejado com a família”. A segunda aborda o conflito do trabalho para a família (tensão) – “Demandas (exigências ou solicitações) do trabalho dificultam o cumprimento de responsabilidades domésticas, como por exemplo, cuidar da casa e dos filhos”. A terceira sobre o conflito da família para o trabalho – “Demandas (exigências ou solicitações) familiares interferem nas responsabilidades profissionais, como por exemplo, chegar pontualmente, cumprir tarefas, não faltar aos compromissos, viajar a trabalho e participar de reuniões fora do horário regular”. A última pergunta avaliou os efeitos simultâneos da família e do trabalho na percepção de falta de tempo para o cuidado pessoal e o lazer – “Demandas (exigências ou solicitações) familiares e profissionais o/a impedem de usar o tempo desejado para seu próprio cuidado e lazer”. As categorias de respostas eram: “muito frequentemente”; “frequentemente”; “às vezes”; “raramente”; “nunca ou quase nunca” ^2016^ . Neste estudo, as opções de resposta foram agrupadas em três categorias: nunca (raramente; nunca ou quase nunca), às vezes e frequentemente (muito frequentemente; frequentemente). 

### Variáveis de Saúde

 Índice de massa corporal (IMC): O peso e a altura foram aferidos por equipe treinada e certificada, por meio de técnica e equipamentos padronizados ^2000^ e o IMC classificado em eutrófico (≤ 24.9 kg/m ^2^ ), sobrepeso (25 kg/m ^2^ e 29.9 kg/m ^2^ ) e obesidade (≥ 30 kg/m ^2^ ). As categorias de baixo peso (≤ 18.5 kg/m ^2^ ) e peso adequado foram agrupadas, devido ao pequeno número de participantes com baixo peso (< 1%) ^2000^ . 

 Indicador do estilo de vida: utilizou-se o indicador proposto e validado por Patrão e colaboradores ^2019^ . Os participantes foram classificados como “menos saudáveis” e “mais saudáveis”. Foram classificados como “menos saudáveis” aqueles que referiram pelo menos dois dos seguintes comportamentos: i- tabagismo atual (“Você fuma cigarros atualmente?”); ii- consumo excessivo de álcool, baseado na quantidade referida do consumo semanal de bebidas alcoólicas em ≥ 210g/semana para os homens e ≥ 140g/semana para as mulheres; iii- inatividade física, mensurada por meio do domínio de atividade física no lazer do Questionário Internacional de Atividade Física (IPAQ) e classificado em < 150 min/semana de exercício físico moderado, atividade ou caminhada e/ou < 60min/semana de atividade física vigorosa ou < 150 min/semana de qualquer combinação de caminhada moderada e atividade física vigorosa); iv - não consumir diariamente frutas, por meio da pergunta “Com que frequência você costuma comer fruta que não seja sob a forma de sucos de fruta?” e v- não consumir diariamente legumes e verduras, por meio da pergunta “Com que frequência você costuma comer verduras ou legumes crus, cozidos ou salteados, outros do que batata, mandioca/mandioca, inhame branco e inhame amarelo?” ^2019^ . 

Comorbidades: Participantes do estudo que relataram pelo menos uma das doenças selecionadas (infarto do miocárdio, acidente vascular cerebral, insuficiência cardíaca, hipertensão ou diabetes) foram classificados como ‘sim’, caso contrário ‘não’.

### Análises estatísticas

 Para a criação das trajetórias de AAS foi utilizada a LCGM, um tipo especial de *Growth Mixture Model* (GMM) que permite a identificação de classes distintas antes da realização do GMM e, por isso, tem sido uma das abordagens recentes mais utilizadas para estudar trajetórias de crescimento. Este modelo leva em consideração as medidas aferidas ao longo do tempo, tendo como objetivo revelar classes latentes distintas, representativas da heterogeneidade dos padrões de trajetórias longitudinais de AAS intrínsecos à população ^2015^ . Dessa maneira, esta técnica permitiu a identificação de classes latentes de trajetórias homogêneas de indivíduos que se assemelham em suas AAS ao longo dos 11 pontos no tempo, baseando-se nas diferenças interindividuais com relação às trajetórias de AAS e aos padrões latentes da população ^2015^ . 

O número de classes latentes adequado foi definido a partir do critério de informação de Akaike (AIC) e o critério de informação Bayesiano (BIC), resultando em três padrões de trajetórias de AAS. Dentro de cada grupo foi avaliada a média de AAS em cada instante no tempo, que variava de 1 (muito bom) até 5 (muito ruim). De acordo com a evolução da média, os padrões de trajetórias foram classificados em “boa”, “regular”, “ruim”.

Foram utilizadas médias, desvios-padrão (DP), valores absolutos (n) e relativos (%) para descrever os grupos de indivíduos quanto às variáveis socioeconômicas, ocupacionais e de saúde. Regressão logística multinomial foi utilizada para estimar as associações entre as variáveis de exposição coletadas na linha de base do estudo (variáveis sociodemográficas, ocupacionais e de saúde) e os padrões de trajetórias de AAS (variável de desfecho), considerando como categoria de referência o padrão de trajetória “boa”. A modelagem foi construída de forma que todas as variáveis estatisticamente significativas no modelo bruto, para ao menos uma das trajetórias, foram testadas no modelo ajustado. O modelo múltiplo foi ajustado por todas as variáveis, sendo mantidas no modelo final aquelas com significância estatística e com contribuição significativa baseada no AIC. Foram estimadas razões de chances (RC), considerando níveis de significância de 5%. As análises foram realizadas no software R, versão 4.0.5, utilizando as bibliotecas “lcmm”, “tidyverse”, “ggplot2” e “factoextra”.

## RESULTADOS

 Os participantes do estudo tinham média de 47 (DP = 6,61)anos de idade, eram em maioria do sexo feminino, se autodeclararam raça/cor da pele branca, tinham escolaridade universitária ou pós-graduação, recebiam mais de três salários mínimos *per capita* e, cerca de 70%, vivam com companheiro(a). A maioria dos participantes foi classificada no trabalho passivo (baixas demandas/baixo controle), trabalhavam 40 horas ou menos por semana e referiram funções não-manual, não-rotineira. Cerca de um terço dos participantes referiram frequentemente o conflito trabalho-família ou a falta de tempo para o autocuidado ou lazer por conta das demandas familiares ou do trabalho. No entanto, referir frequentemente o conflito da família para o trabalho foi menos comum (6,7%). Mais de 60% dos participantes tinham sobrepeso ou obesidade, ou apresentavam comorbidades, e quase 30% foram classificados com estilo de vida menos saudável ( Tabela 1 ). 

**Tabela 1.** Descrição da amostra segundo as variáveis utilizadas no estudo. Estudo Longitudinal de Saúde do Adulto (ELSA-Brasil, 2008–2020).

Variáveis da linha de base	Total (n = 7.738)
Idade m (DP)	47,7 (6,61)
Sexo n (%)	
Feminino	4.101 (53,0)
Masculino	3.637 (47,0)
Raça/cor autodeclarada n (%)	
Branca	4.114 (53,2)
Parda	2.344 (30,3)
Preta	1.280 (16,5)
Escolaridade n (%)	
Pós-graduação	2.947 (38,0)
Universitário completo	1.358 (17,6)
Ensino médio completo	3.433 (44,4)
Renda em salários mínimos *per capita* n (%)	
≤ 3 Salários	3.818 (49,3)
> 3 Salários	3.920 (50,7)
Situação conjugal n (%)	
Com companheiro	5.307 (68,6)
Sem companheiro	2.341 (31,4)
Estresse no trabalho n (%)	
Baixo desgaste no trabalho	1.718 (22,2)
Trabalho ativo	1.367(17,7)
Trabalho passivo	3.031 (39,2)
Alto desgaste no trabalho	1.622 (20,9)
Apoio social no trabalho n (%)	
Alto	3.427 (44,3)
Baixo	4.311 (55,7)
Carga horária semanal de trabalho n (%)	
≤ 40 horas	5.060 (65,4)
> 40 horas	2.678 (34,6)
Natureza da ocupação n (%)	
Manual	1.251 (16,2)
Não-manual rotineiro	2.213 (28,6)
Não-manual, não rotineiro	4.274 (55,2)
Conflito trabalho-família relacionado ao tempo n (%)	
Nunca	3.029 (39,1)
Às vezes	2.375 (30,7)
Frequentemente	2.334 (30,2)
Conflito trabalho-família relacionado à tensão n (%)	
Nunca	3.741 (48,3)
Às vezes	2.365 (30,6)
Frequentemente	1.632 (21,1)
Conflito família-trabalho n (%)	
Nunca	5.168 (66,8)
Às vezes	2.049 (26,5)
Frequentemente	521 (6,7)
Falta de tempo para o autocuidado e lazer n (%)	
Nunca	2.817 (36,4)
Às vezes	2.550 (33,0)
Frequentemente	2.371 (30,6)
Índice de massa corporal n (%)	
Eutrófico	3.003 (38,8)
Sobrepeso	3.074 (39,7)
Obesidade	1.661 (21,5)
Indicador de estilo de vida n (%)	
Saudável	5.427 (70,1)
Menos saudável	2.311 (29,9)
Presença de comorbidades n (%)	
Não	2.521 (32,6)
Sim	5.217 (67,4)

DP: desvio padrão.

**Figura 2.**
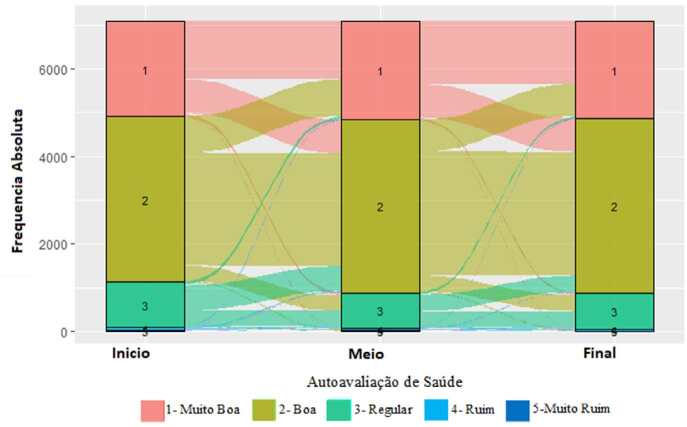
Distribuição e transições da autoavaliação de saúde nos momentos inicial (ponto 1), mediano (ponto 6) e final (ponto 11) do período de acompanhamento. Estudo Longitudinal de Saúde do Adulto (ELSA–Brasil, 2008–2020).

 O comportamento da AAS ao longo do tempo se mostrou estável, sendo que a maioria das transições ocorreram entre as categorias imediatamente anterior ou posterior. As transições na categoria 3 (regular) foram mais equilibradas entre permanência ou transição para a categoria 2 (boa). Observou-se também uma frequência muito baixa de ocorrências para as categorias 4 (ruim) e 5 (muito ruim) em todos os momentos observados ( Figura 2 ). 

Nota: número de participantes com autoavaliação de saúde muito boa, boa, regular, ruim e muito ruim, em cada tempo, foi respectivamente de: início – 2.356, 4.131, 1.144, 89, 18; meio – 2.380, 4.207, 887, 63, 15; e final – 2.262, 4.084, 847, 52, 5.

**Figura 3.**
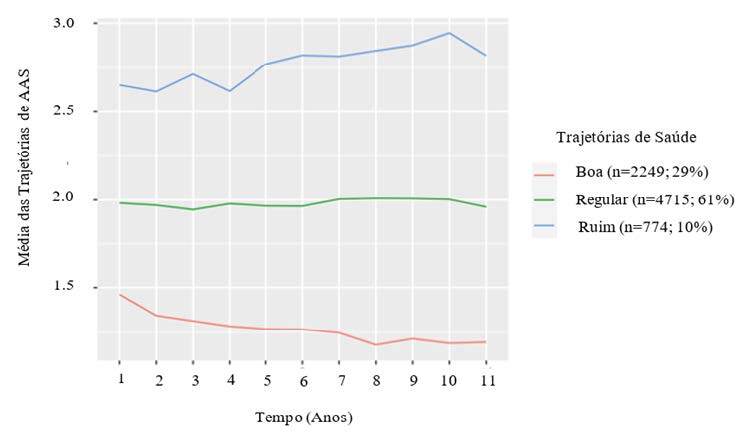
Média da autoavaliação de saúde (AAS) por cada padrão de trajetória identificado nos 11 anos de seguimento. Estudo Longitudinal de Saúde do Adulto (ELSA–Brasil, 2008–2020 – n = 7.738).

 Após aplicação do LCGM, os participantes foram classificados em três padrões de evolução de AAS relativamente estáveis no período avaliado, sendo que as maiores médias representam pior autoavaliação de saúde ( Figura 3 ). O padrão 1 (n = 2.249; 29%), denominado “boa”, incluiu participantes consistentemente positivos sobre sua saúde ao longo do tempo (Média = 1,42; DP = 0,57 pontos). O padrão 2 (n = 4.715; 61%), denominado “regular”, apresentou maior frequência, compreendendo as pessoas que avaliaram sua saúde de forma menos positiva que os do padrão 1 (Média = 1,96; DP = 0,61 pontos). O padrão 3 (n = 774; 10%), denominado “ruim”, foi composto por pessoas que apresentaram a AAS de forma menos positiva que os padrões 1 e 2, respectivamente (Média = 2,62; DP = 0,74 pontos). Dentro de cada padrão, os respondentes exibiram alguma variabilidade na AAS, tendo leve tendência de piora nos padrões 1 e 2 ao longo do tempo avaliado ( Figura 3 ). 

 No que se refere ao padrão de AAS regular, os modelos de regressão logística multinomial, ajustados por todas as demais variáveis, mostraram que chances mais elevadas foram observadas entre os que tinham menor renda e aqueles com ensino médio ou universitário, comparado a aqueles com pós-graduação. Quanto aos aspectos do trabalho, alto desgaste no trabalho e trabalho passivo (comparado ao trabalho de baixo desgaste), além de baixo apoio social aumentaram as chances de pertencer ao padrão de AAS regular, enquanto a carga horária > 40 horas/semana se mostrou protetora. Além disso, os participantes com estilo de vida menos saudável, com sobrepeso ou obesidade e comorbidades apresentaram maiores chances de pertencer ao padrão de AAS regular, comparado ao padrão de trajetória boa ( Tabela 2 ). 

 Para o padrão de trajetória de AAS ruim, os modelos de regressão ajustados evidenciaram maiores chances (em torno de 30%) para as mulheres, aqueles que se autodeclararam pardos, com menor renda, que referiram baixo apoio social no trabalho, conflito frequente do trabalho para a família relacionado à tensão e ao conflito às vezes da família para o trabalho. Chances 50% mais elevadas de pertencer ao padrão de AAS ruim foram observadas entre aqueles que estudaram até o ensino médio, aqueles classificados no trabalho passivo, que referiram conflito frequente da família para o trabalho e aqueles classificados com estilo de vida menos saudável. Além disso, chances ainda mais elevadas (em torno de 75%) de pertencer ao padrão de trajetória de AAS ruim foram observadas para os classificados no alto desgaste no trabalho e com sobrepeso. Chances em torno de 80% de padrão de AAS ruim também foram observadas para aqueles que referiram frequentemente falta de tempo para o autocuidado e lazer. Os trabalhadores manuais e aqueles com comorbidades tiveram o dobro de chance de pertencer ao padrão ruim. A presença de obesidade aumentou em quatro vezes a chance de pertencer ao padrão de AAS ruim. De forma semelhante ao observado na trajetória regular, a carga horária > 40 horas/semana se mostrou também protetora ( Tabela 2 ). 

**Tabela 2.** Razão de chances e respectivos intervalos com 95% de confiança dos modelos de regressão logística multinomial na associação entre fatores sociodemográficos, comportamentais e ocupacionais e os padrões de trajetórias de autoavaliação de saúde. Estudo Longitudinal de Saúde do Adulto (ELSA-Brasil, 2008–2020).

Variáveis da linha de base	**Padrão de trajetória regular**	**Padrão de trajetória regular**	**Padrão de trajetória ruim**	**Padrão de trajetória ruim**
	Modelo bruto RC (IC95%)	Modelo ajustado RC (IC95%)	Modelo bruto RC (IC95%)	Modelo ajustado RC (IC95%)
Idade	1,00 (0,99–1,01)	1,00 (0,99–1,01)	**1,01 (1,00–1,03)**	**1,01 (1,00–1,03)**
Sexo				
Masculino	**1**	1	**1**	1
Feminino	0,94 (0,85–1,04)	1,01 (0,90–1,13)	**1,28 (1,08–1,51)**	**1,31 (1,08–1,60)**
Raça/cor autodeclarada				
Branca	1	1	1	1
Parda	**1,29 (1,15–1,45)**	1,02 (0,90–1,16)	**1,94 (1,61–2,35)**	**1,32 (1,08–1,62)**
Preta	**1,41 (1,22–1,64)**	0,92 (0,79–1,09)	**2,53 (2,03–3,16)**	1,20 (0,94–1,54)
Escolaridade				
Pós-graduação	1	1	1	1
Universitário	**1,58 (1,37–1,83)**	**1,33 (1,13–1,55)**	**1,47 (1,12–1,91)**	1,05 (0,78–1,40)
Até ensino médio	**2,09 (1,87–2,34)**	**1,25 (1,04–1,50)**	**3,55 (2,94–4,29)**	**1,53 (1,13–2,08)**
Salário mínimo *per capita*				
> 3 salários mínimos	1	1	1	1
≤ 3 salários mínimos	**1,81 (1,63–2,00)**	**1,16 (1,02–1,33)**	**3,00 (2,53–3,56)**	**1,37 (1,09–1,72)**
Situação conjugal				
Sem companheiro (a)	1	1	1	1
Com companheiro(a)	1,10 (0,98–1,22)	1,07 (0,95–1,20)	**0,83 (0,70–0,99)**	**0,86 (0,72–1,05)**
Estresse no trabalho				
Baixo desgaste	1	1	1	1
Trabalho ativo	0,91 (0,78–1,06)	0,87 (0,74–1,03)	1,30 (0,98–1,74)	1,06 (0,78–1,45)
Trabalho passivo	**1,59 (1,39–1,81)**	**1,19 (1,03–1,38)**	**2,56 (2,01;3,26)**	**1,49 (1,15–1,94)**
Alto desgaste	**1,81 (1,55–2,12)**	**1,32 (1,11–1,57)**	**3,47 (2,66–4,53)**	**1,75 (1,31–2,35)**
**Apoio social no trabalho**				
Alto	1	1	1	1
Baixo	**1,12 (1,02–1,24)**	**1,18 (1,06–1,31)**	**1,33 (1,12–1,57)**	**1,36 (1,13–1,63)**
**Carga horária de trabalho**				
≤ 40 horas/semana	1	1	1	1
> 40 horas/semana	**0,74 (0,67–0,82)**	**0,85 (0,75–0,96)**	**0,64 (0,53–0,76)**	**0,74 (0,61–0,91)**
**Natureza da ocupação**				
Não manual, não rotineiro	1	1	1	1
Trabalho rotineiro não manual	**1,58 (1,41–1,78)**	**1,20 (1,03–1,39)**	**2,23 (1,84–2,70)**	1,21 (0,94–1,55)
Trabalho manual	**2,43 (2,06–2,86)**	**1,63 (1,32–2,02)**	**4,20 (3,34–5,29)**	**2,08 (1,53–2,84)**
**Conflito trabalho para família relacionado ao tempo**				
Nunca	1	1	1	1
Às vezes	1,00 (0,88–1,12)	-	0,98 (0,80–1,20)	**-**
Frequentemente	**0,85 (0,76–0,96)**	-	1,02 (0,84–1,25)	**-**
**Conflito trabalho para família relacionado à tensão**				
Nunca	1	1	1	1
Às vezes	0,95 (0,84–1,06)	1,02 (0,89–1,16)	1,05 (0,87–1,28)	1,10 (0,89–1,37)
Frequentemente	1,07 (0,94–1,22)	1,16 (0,99–1,37)	**1,49 (1,21–1,83)**	**1,39 (1,08–1,80)**
**Conflito família para trabalho**				
Nunca	1	1	1	1
Às vezes	1,11 (0,99–1,25)	1,12 (0,99–1,27)	**1,31 (1,08–2,57)**	**1,31 (1,07–1,61)**
Frequentemente	**1,35 (1,08–1,68)**	1,22 (0,97–1,55)	**2,09 (1,54–2,83)**	**1,58 (1,13–2,22)**
**Falta de tempo para autocuidado e lazer**				
Nunca	1	1	1	1
Às vezes	1,06 (0,94–1,20)	**1,18 (1,03–1,35)**	1,12 (0,91–1,37)	1,25 (0,99–1,58)
Frequentemente	1,07 (0,95–1,22)	**1,27 (1,08–1,49)**	**1,53 (1,25–1,87)**	**1,82 (1,41–2,34)**
**Índice de Massa Corporal**				
Eutrófico	1	1	1	1
Sobrepeso	**1,65 (1,48–1,85)**	**1,50 (1,34–1,69)**	**2,02 (1,65–2,48)**	**1,73 (1,40–2,13)**
Obesidade	**2,81 (2,41–3,27)**	**2,29 (1,95–2,69)**	**6,25 (5,00–7,82)**	**4,25 (3,35–5,39)**
**Indicador de estilo de vida**				
Saudável	1	1	1	1
Menos saudável	**1,58 (1,41–1,78)**	**1,36 (1,21–1,54)**	**2,01 (1,68–2,39)**	**1,57 (1,30–1,89)**
**Presença de comorbidade**				
Não	1	1	1	1
Sim	**1,98 (1,76–2,22)**	**1,63 (1,43–1,85)**	**3,28 (2,76–3,90)**	**2,26 (1,86–2,75)**

RC: razão de chances; IC95%: intervalo de confiança de 95%.

Nota: em negrito aquelas com associações significativas ao nível de 5% de significância.

Padrão de trajetória de referência = boa. O modelo múltiplo foi ajustado por todas as variáveis, sendo mantidas no modelo final aquelas com significância estatística e com contribuição significativa ao modelo. A variável “conflito trabalho–família (tempo) não foi mantida no modelo final ajustado, pois a sua retirada contribuiu para o melhor ajuste do modelo.

## DISCUSSÃO

 Os três padrões de trajetórias de evolução da AAS que emergiram por meio da aplicação de LCGM mostraram-se relativamente estáveis, com leve tendência de piora nos padrões de trajetórias de AAS ruim e boa ao longo do tempo. Este resultado é compatível com outros estudos internacionais sobre trajetórias de saúde, que também evidenciaram que a maioria das pessoas apresentam trajetórias estáveis ao longo do tempo, mas que existem grupos menores com trajetórias de declínio e de melhora ^4^^,^^2020^^,^^2012^ . 

No presente estudo, cerca de 10% dos entrevistados seguiram uma trajetória de AAS ruim consistente ao longo do tempo avaliado. Na avaliação das características associadas a trajetória ruim, o sexo feminino, as piores condições socioeconômicas e ocupacionais, assim como o estilo de vida menos saudável e a presença de comorbidades se destacaram. Características semelhantes, porém com chances atenuadas, foram observadas no padrão de trajetória de AAS regular.

 No que se refere às variáveis sociodemográficas, sexo feminino, raça/cor parda, menor escolaridade e menor renda, estiveram associadas aos piores padrões de trajetórias de AAS. A associação das variáveis escolaridade e renda com os padrões de trajetórias evidencia o quanto as desigualdades podem afetar a saúde da população. Nessa perspectiva, a Pesquisa Nacional por Amostra de Domicílios (2016) ^2022^ mostrou que as populações preta e parda têm menor escolaridade e, quando empregadas, geralmente recebem metade da renda, comparadas à população branca ^2017^ . Importante destacar que nas análises de regressão simples, pretos também tiveram maiores chances de AAS ruim e regular. É provável que o ajuste pela escolaridade e/ou renda tenham contribuído para que a associação entre pretos e AAS ruim perdesse a significância estatística nos modelos múltiplos, visto que renda e escolaridade podem ser mediadores da associação entre raça e AAS. 

 A associação de piores condições socioeconômicas com piores padrões de trajetórias de AAS também foi encontrada em outros estudos ^2022^^,^^8^ . Um estudo recente realizado no Reino Unido evidenciou que o aumento na renda impacta positivamente as trajetórias de autoavaliação de saúde ^2022^ . As condições socioeconômicas influenciam a saúde de diferentes formas, seja na aquisição de bens e serviços de saúde, no acesso aos serviços de saúde ou nas condições de moradia, alimentação e estilo de vida ^2022^^,^^2021^ . Dessa maneira, tais condições estão estreitamente interligadas, impactando a saúde. 

 Embora seja esperada uma relação direta entre o envelhecimento e uma pior autoavaliação de saúde, no presente artigo não foram observadas associações entre idade e padrões de trajetórias de AAS. Cabe destacar que esta amostra é composta apenas por trabalhadores ativos em todo o período de seguimento do estudo, o que pode ter contribuído para a redução do efeito da idade ^2017^ , dado que os mais velhos foram excluídos com base no critério da aposentadoria. 

 Diferentemente dos estudos que abordam a associação das trajetórias de AAS apenas com fatores socioeconômicos ou de saúde, o presente estudo fornece uma contribuição importante para a literatura, ao incluir também a avaliação da associação da ASS com fatores ocupacionais. Em tal abordagem, o trabalho passivo ou de alto desgaste, o baixo apoio social, a ocupação manual, o conflito do trabalho para a família ou da família para o trabalho e a falta de tempo para o autocuidado e lazer foram associados aos piores padrões de trajetórias de saúde. As características ocupacionais relacionadas ao estresse no trabalho, tipo de ocupação e apoio social no trabalho também foram associadas às piores trajetórias de AAS em outros dois estudos ^2010^^,^^2006^ . Estudo brasileiro ^2021^ identificou que pessoas com trabalho ativo, passivo e de alto desgaste têm menor risco de mudar a AAS de regular para boa. O estudo também verificou que o grupo com baixo apoio social no trabalho tem menos chance de migrar da AAS ruim para boa ao longo do tempo. 

 Em conjunto, os achados reforçam a concepção de que os aspectos do trabalho podem impactar a saúde dos trabalhadores de diferentes formas. O baixo *status* ocupacional, a alta demanda e o baixo controle no trabalho estão diretamente relacionados à ocorrência de estresse laboral. Além disso, o baixo apoio social no trabalho colabora para acentuar os efeitos do estresse no trabalho ^2017^ , aumentando o risco de adoecimento físico e psíquico ^2022^ . 

 Além dos aspectos do ambiente de trabalho, o conflito entre demandas do trabalho e familiares associou-se aos piores padrões de trajetórias de AAS. Alguns estudos abordam a associação do conflito trabalho-família com a AAS ruim, sendo encontradas piores associações em mulheres ^2010^^,^^2017^ . Estudo que abordou a relação do conflito com as trajetórias de AAS evidenciou aqueles com menor nível educacional que relataram ter exaustão no trabalho tinham maiores chances de ter trajetórias de saúde classificadas como “ruim”, se comparadas às pessoas que têm maior nível educacional ^2014^ . 

 Em relação à jornada de trabalho, nossos resultados apontaram que trabalhar mais de 40 horas/semana se mostrou protetor para o pior padrão de trajetórias de saúde. Estudo longitudinal realizado na Coreia evidenciou que longas jornadas de trabalho (> 52 horas) estão associadas com a piora da AAS ao longo do tempo para ambos os sexos. No entanto, os autores também verificaram que, somente entre as mulheres ^2018^ , trabalhar menos de 40 horas semanais associou-se às piores trajetórias de saúde. Ainda não existe um consenso sobre a associação da jornada de trabalho com a AAS. Nossa hipótese para os resultados encontrados refere-se ao fato de que as longas jornadas podem estar associadas a AAS boa, uma vez que pessoas mais saudáveis conseguem trabalhar por mais horas semanais. Considerando que a maioria das pessoas apresentam trajetórias estáveis ao longo do tempo ^4^^,^^2020^^,^^2012^ , é possível que as jornadas mais curtas sejam um reflexo da saúde ruim. Portanto, as associações parecem diferir de acordo com o tipo de estudo, a população e o sexo ^2018^^,^^2011^ . 

 O fato de aspectos da saúde, como obesidade, estilo de vida menos saudável e presença de comorbidades, também estarem associados ao pior padrão de trajetórias de AAS está em concordância com outros estudos ^4^^,^^2012^ que observaram associações de hábitos de vida não saudáveis e dieta inadequada com as piores trajetórias de saúde. A relação entre obesidade e os piores padrões de trajetória de AAS pode ser explicada por comorbidades relacionadas à obesidade, dado que a obesidade está associada a uma série de doenças. Além disso, está relacionada a comportamentos de saúde menos saudáveis, o que também pode levar a uma qualidade de vida ruim e, consequentemente, a uma pior AAS ^2020^ . Além disso, a tendência de as pessoas com obesidade avaliarem a sua saúde de forma negativa pode estar relacionado ao aumento de informações sobre as consequências negativas disso para a sua saúde ^2016^ . Vale destacar que estes comportamentos não têm todos necessariamente a mesma importância na promoção da saúde (ex. fumar diariamente é mais prejudicial à saúde do que não comer frutas diariamente) e aqui foram avaliados como tal. No entanto, este indicador é uma tentativa, ainda que simplista, de analisar os comportamentos em simultâneo, que é mais próximo do que define o estilo de vida na realidade das pessoas. 

 O presente estudo se destaca por tratar da identificação de padrões de trajetórias de AAS pelo método de agrupamento de curva de crescimento de classe latente, o que ainda é pouco utilizado, e por analisar a AAS ao longo de 11 pontos no tempo pela primeira vez na população brasileira. Cabe ressaltar que esta pesquisa incluiu a análise de variáveis relacionadas ao estilo de vida, como recomendado em recente estudo ^6^ . Além disso, a população estudada compreendeu trabalhadores ativos de uma grande coorte da América Latina, o que oferece oportunidades raras de investigação, como a avaliação das características ocupacionais, além dos fatores socioeconômicos e de saúde já explorados na literatura. 

Quanto às limitações, a generalização dos achados deve ser realizada com cautela, uma vez que os resultados se referem a uma coorte de servidores públicos. A estabilidade dos participantes no emprego e na renda pode ter influenciado a baixa proporção de AAS ruim e muito ruim. Outra limitação se refere às variáveis de exposição avaliadas apenas na linha de base. No entanto, entendemos que a alta estabilidade dos servidores pode ter atenuado o efeito da variabilidade, sobretudo de características sociodemográficas. Por fim, apesar de o estudo ter utilizado dados autorreferidos, que podem estar sujeitos a vieses, foram adotados instrumentos validados e rigoroso processo de garantia e controle de qualidade ao longo de todas as fases.

## CONCLUSÕES

O presente artigo contribui para o entendimento dos fatores associados aos padrões de trajetórias de autoavaliação de saúde em trabalhadores ativos, o que ainda é escasso na literatura. Os resultados apontaram três padrões de trajetórias, sendo que aqueles mais adversos estavam relacionados às piores condições socioeconômicas e ocupacionais, mesmo após o ajuste por variáveis mais proximais, tais como o estilo de vida, o excesso de peso e as comorbidades. Os resultados reforçam a importância da elaboração de políticas públicas que visem minimizar as desigualdades sociais e aumentar a promoção da saúde na população brasileira, fatores amplamente reconhecidos. Além disso, o estudo inova ao incluir variáveis relacionadas ao trabalho, apontando para a necessidade de políticas que promovam um ambiente de trabalho saudável, aliado ao equilíbrio entre as demandas do trabalho e da vida pessoal, fatores com grande potencial de intervenção, sobretudo no cenário atual de adiar cada vez mais a aposentadoria e manter os trabalhadores ativos por mais tempo.
