# Supplementary material for: Padrões de trajetórias de autoavaliação de saúde e fatores associados no ELSA-Brasil
Source: Rev Saude Publica. 2024 Dec 16;58:50. [Article in Portuguese] doi: 10.11606/s1518-8787.2024058005580 (PMC11655056; doi:10.11606/s1518-8787.2024058005580)
Supplement: Translation

# Translation: Patterns of self-rated health trajectories and associated factors in ELSA-Brasil

**Published:** 2024-12-16

**Authors:** Camila Arantes Ferreira Brecht D’Oliveira, Daniela Polessa Paula, Aline Silva-Costa, Odaleia Barbosa de Aguiar, Lidyane V. Camelo, Ana Luísa Patrão, Maria de Jesus Mendes da Fonseca, Rosane Härter Griep

**Affiliations:** I Ministério da Saúde. Secretaria de Vigilância em Saúde e Ambiente. Departamento de Análise Epidemiológica e Vigilância de Doenças Crônicas Não Transmissíveis. Brasília, DF, Brasil; II Instituto Brasileiro de Geografia e Estatística. Escola Nacional de Ciências Estatísticas. Rio de Janeiro, RJ, Brasil; III Universidade do Estado do Rio de Janeiro. Instituto de Matemática e Estatística. Rio de Janeiro, RJ, Brasil; IV Universidade Federal do Triângulo Mineiro. Instituto de Ciências da Saúde. Departamento de Saúde Coletiva. Uberaba, MG, Brasil; V Universidade do Estado do Rio de Janeiro. Instituto de Nutrição. Departamento de Nutrição Aplicada. Rio de Janeiro, RJ, Brasil; VI Universidade Federal de Minas Gerais. Faculdade de Medicina. Departamento de Medicina Preventiva e Social. Belo Horizonte, MG, Brasil; VII Universidade do Porto. Faculdade de Psicologia e de Ciências da Educação. Centro de Psicologia da Universidade do Porto. Porto, Portugal; VIII Fundação Oswaldo Cruz. Escola Nacional de Saúde Pública Sérgio Arouca. Departamento de Epidemiologia e Métodos Quantitativos. Rio de Janeiro, RJ, Brasil; IX Fundação Oswaldo Cruz. Instituto Oswaldo Cruz. Laboratório de Educação em Ambiente e Saúde. Rio de Janeiro, RJ, Brasil

**Keywords:** Health Trajectories, Self-rated Health, Latent Class Analysis, Brazil

## Abstract

To describe patterns of self-rated health (SRH) trajectories and investigate their association with sociodemographic, occupational, and health factors.

The sample consisted of 7,738 active public servants from the Brazilian Longitudinal Study of Adult Health (ELSA-Brasil), evaluated from 2008 to 2020. The patterns of SRH trajectories were obtained by eleven time points, using the latent class growth curve. A multinomial logistic model was used to test associations between the exposures and patterns of trajectories of SRH.

Three patterns of trajectories of SRH were identified: i- good, ii- moderate, and iii- poor (29%, 61%, and 10% of the participants, respectively). Adjusted results showed that women, mixed-race, frequent work to family or family to work conflict were associated with a greater chance of poor pattern of trajectory of SRH, compared to good pattern. Besides, high school, low income, passive work, high strain, low social support, lack of time selfcare and leisure, overweight, obesity, unhealthy lifestyle, and the presence of comorbidities were associated with a greater chance of moderate and poor pattern of trajectory of SRH, when compared with a good pattern.

Adverse socioeconomic and occupational conditions, as well as unhealthy lifestyle and comorbidities were associated with worse SRH patterns of trajectories.

## INTRODUCTION

 Self-rated health (SRH) is an indicator based on the individual perspective, it is widely used in the assessment of global health ^1^^,^^2^ and considered a good predictor of mortality ^2^^,^^3^ . The association of SRH with morbidity and mortality is quite widespread, with 80% agreement with clinical assessment of the presence or absence of a chronic health condition ^1^^–^^3^ . In addition to these aspects, SRH is also considered to be an easy-to-apply, accurate, and low-cost indicator ^1^^–^^3^ . 

 In epidemiological studies, SRH has predominantly been measured at a single point in time ^3^^,^^4^ . In a study on mortality ^3^ , the predictive capacity of SRH at a single point in time showed a time-dependent effect, i.e., it was attenuated over time ^3^ . Knowing the trajectories of SRH makes it possible to distinguish people’s health status consistently or intermittently over time ^5^ , which is useful for targeting disease prevention efforts ^6^ . 

 Studies analyzing SRH over time are more recent, identifying its sharpest decline among females, older people with low levels of education and income ^4,6–8^ , as well as among those with a lifestyle marked by smoking, alcohol consumption, sedentary lifestyles or low levels of leisure-time physical activity, fruit and vegetable consumption, or with multiple chronic health conditions ^4^^–^^9^ . 

 Regarding occupational factors associated with SRH over time, international literature shows that repetitive work, of high psychological demand, low social support ^2000^ , and job-related insecurity ^2019^ are associated with declines in SRH. For workers in good health before retirement, low occupational status (maintenance, cleaning, and construction workers, among others), physically strenuous work, and job strain were associated with a higher risk of SRH decline during the transition to retirement ^2020^ . In Brazil, as far as we could find, only one article investigated the relationship between occupational factors and changes in SRH between two time points, during 10 years of follow-up, using the Markov multistate model ^2020^ . The results of this study showed that people who perform passive work (a classification that combines low psychological demands with low control at work) or high-demand work (a classification that combines high psychological demands with low control at work) have a lower risk of transitioning from fair to good SRH ^2021^ . 

This study aims to describe patterns of SRH trajectories over 11 years of follow-up, as well as to investigate sociodemographic, occupational, and health factors associated with patterns of SRH trajectories in a Brazilian cohort.

## METHODS

### Study Design and Participants

 The Brazilian Longitudinal Study of Adult Health (ELSA-Brasil), a multicenter cohort of 15,105 active and retired civil servants aged from 35 to 74, covers six public higher education and research institutions in Brazil ^2021^ . Data is collected from in-person examinations, interviews, and annual telephone follow-up. All waves of the study follow standardized procedures and are conducted by duly trained and certified staff ^14–15^ . Participants who were active in both the baseline (2008–2010) and second (2012–2014) and third waves of the study (2017–2019) were included in this study. They were interviewed through annual telephone monitoring (2009 until 28/12/2020). 

 We excluded (i) retirees (n = 6,470), both because of the lack of occupational information on this group at baseline and because those who retired during follow-up had a different health behavior profile from active workers; (ii) deaths (n = 69); (iii) participants who declared themselves as Yellow (n = 198) or Indigenous (n = 74), given the small number of participants in each category; and (iv) those who did not respond to any of the variables of interest in this study (n = 556). The final sample, considering active workers throughout the study follow-up period, was made up of 7,738 participants ( Figure 1 ). 

**Figure 1.** Flowchart of the participants included in the analyses. Brazilian Longitudinal Study of Adult Health (ELSA-Brasil 2008-2020).

The study was approved by the research and ethics committees of the institutions involved and all participants signed an informed consent form.

## STUDY VARIABLES

### Outcome: Self-rated health (SRH)

Both in the three waves of the study and in the annual follow-up calls, the following question was asked: “In general, compared with other people your age, how would you rate your health?” The answer options were: “very good,” “good,” “fair,” “poor,” and “very poor.” In this way, each participant’s self-assessment could vary from 1 (very good) to 5 (very poor), at 11 points in time (three waves and eight annual follow-up calls) during the study period (2008 to 2020). After applying the latent class growth model (LCGM), described in detail in the data analysis section, three patterns of SRH trajectories were identified, called “good,” “fair,” and “poor.”

## EXPOSURE VARIABLES

For this study, the socioeconomic, occupational, and health variables measured at baseline were evaluated.

### Sociodemographic variables

 Age (continuous); gender (male; female); self-reported race/skin color (White; Brown [Mixed-race]; Black); schooling (up to complete high school; college; postgraduate); net family income *per capita* , divided into salaries based on the 2008 minimum wage of R$ 415.00 (≤ 3 minimum wages; > 3 minimum wages); marital status (with a partner; without a partner). 

### Occupational variables

 Stress at work: measured using the Brazilian version of the Swedish Demand Control Support Questionnaire (DCSQ), it covers psychological demand, control, and social support in the workplace. In this study, the item on repetitive work was removed, considering the analysis of the dimensional structure of the questionnaire in the Brazilian context ^2010^ . DCSQ scores were summed and dichotomized into high and low, using the median of the dimensions as a cut-off point (demand = 14; control = 17; support = 20). The work stress variable was categorized into quadrants: low job stress (low psychological demand/high control); active job (high psychological demand/high control); passive job (low psychological demand/low control); and high job stress (high psychological demand/low control). Social support was categorized as high and low based on the median of the distribution of scores. 

Working hours: classified as ≤ 40 hours/week and > 40 hours/week.

Nature of Occupation: variable obtained through the following question: “Please describe the main activities you carry out in your day-to-day work at (name of institution).” The classification of occupations, according to the nature of the tasks required to carry them out, considered the appropriate skills for performing Manual (or not) and routine (or not) tasks, in four categories: non-routine non-Manual, routine non-Manual, non-routine Manual, routine Manual. In this study, the non-routine Manual and routine Manual categories were grouped under “Manual.”

 Work-family conflict. Work-family conflict was measured by four questions ^2016^ . The first addresses the conflict from work to family (time) – “Work demands (requirements or requests) prevent you from spending the desired amount of time with your family.” The second addresses the conflict from work to family (strain) – “Demands (requirements or requests) from work make it difficult to fulfill domestic responsibilities, such as looking after the house and children.” The third question asked about the conflict from family to work – “Family demands interfere with professional responsibilities, such as arriving on time, fulfilling tasks, not missing appointments, traveling for work and attending meetings outside of regular hours.” The last question assessed the Yesultaneous effects of family and work on the perception of lack of time for personal care and leisure – “Family and professional demands prevent you from using the time you want for self-care and leisure.” The response categories were: “very often”; “often”; “sometimes”; “rarely”; “never or almost never” ^2016^ . In this study, the response options were grouped into three categories: never (rarely; never or almost never), sometimes and often (very often; often). 

### Health Variables

 Body mass index (BMI): Weight and height were measured by a trained and certified team using standardized equipment and techniques ^2000^ and BMI was classified as eutrophic (≤ 24.9 kg/m ^2^ ), overweight (25 kg/m ^2^ and 29.9 kg/m ^2^ ) and obese (≥ 30 kg/m ^2^ ). The categories of underweight (≤ 18.5 kg/m ^2^ ) and adequate weight were grouped together due to the small number of underweight participants (< 1%) ^2000^ . 

 Lifestyle indicator: The indicator proposed and validated by Patrão et al. ^2019^ was used. Participants were classified as “less healthy” and “healthier.” Those who reported at least two of the following behaviors were classified as “less healthy”: i- current smoking (“Do you currently smoke cigarettes?”); ii- excessive alcohol consumption, based on reported weekly alcohol consumption of ≥ 210g/week for men and ≥ 140g/week for women; iii- physical inactivity, measured using the leisure-time physical activity domain of the International Physical Activity Questionnaire (IPAQ) and classified as < 150 min/week of moderate physical exercise, activity or walking and/or < 60 min/week of vigorous physical activity or < 150 min/week of any combination of moderate walking and vigorous physical activity); iv - not consuming fruit on a daily basis, using the question “How often do you eat fruit other than in the form of fruit juice?”; and v - not consuming legumes and vegetables on a daily basis, with the question “How often do you usually eat raw, boiled or sauteed legumes or vegetables other than potatoes, cassava/manioc, white yams and yellow yams?” ^2019^ . 

Comorbidities: Study participants who reported at least one of the selected diseases (myocardial infarction, stroke, heart failure, hypertension, or diabetes) were classified as “yes,” otherwise “no.”

### Statistical analysis

 To create the SRH trajectories we used LCGM, a special type of Growth Mixture Model (GMM) that allows distinct classes to be identified before the GMM is carried out and has therefore been one of the most widely used recent approaches to studying growth trajectories. This model considers the measures taken over time to reveal distinct latent classes, representative of the heterogeneity of the longitudinal trajectory patterns of SRH intrinsic to the population ^2015^ . Thus, this technique allowed the identification of latent classes of homogeneous trajectories of individuals who resemble each other in their SRH over 11 points in time, based on inter-individual differences in relation to SRH trajectories and latent patterns of the population ^2015^ . 

The appropriate number of latent classes was defined using the Akaike information criterion (AIC) and the Bayesian information criterion (BIC), resulting in three patterns of SRH trajectories. Within each group, the mean SRH at each moment in time was evaluated, ranging from 1 (very good) to 5 (very poor). According to the evolution of the mean, the trajectory patterns were classified as “good,” “fair,” or “poor.”

Means, standard deviations (SD), absolute values (n) and relative values (%) were used to describe the groups of individuals in terms of socioeconomic, occupational, and health variables. Multinomial logistic regression was used to estimate the associations between the exposure variables collected at the study baseline (sociodemographic, occupational, and health variables) and the patterns of SRH trajectories (outcome variable), considering the “good” trajectory pattern as the reference category. The modeling was constructed in such a way that all statistically significant variables in the crude model, for at least one of the trajectories, were tested in the adjusted model. The multiple model was adjusted for all the variables, and those with statistical significance and a significant contribution based on the AIC were kept in the final model. Odds ratios (OR) were estimated, considering significance levels of 5%. The analyses were carried out in the R software, version 4.0.5, using the “lcmm,” “tidyverse,” “ggplot2,” and “factoextra” libraries.

## RESULTS

 The study participants had a mean age of 47 (6.61) years, were mostly female, self-declared as of white race/skin color, had college or postgraduate education, earned more than three minimum wages *per capita,* and about 70% lived with a partner. The majority of participants were classified as working passively (low demands/low control), worked 40 hours or less per week and reported non-Manual, non-routine duties. Around a third of the participants frequently reported work-family conflict or a lack of time for self-care or leisure due to family or work demands. However, frequently mentioning family-to-work conflict was less common (6.7%). More than 60% of the participants were overweight or obese, or had comorbidities, and almost 30% were classified as having a less healthy lifestyle (Table 1). 

**Table 1.** Description of the sample according to the variables used in the study. Brazilian Longitudinal Study of Adult Health (ELSA-Brasil, 2008-2020).

Baseline variables	Total (n = 7.738)
Age m (SD)	47.7 (6.61)
Sex n (%)	
Female	4.101 (53.0)
Male	3.637 (47.0)
Self-declared race/color n (%)	
White	4.114 (53.2)
Brown/Mixed-race	2.344 (30.3)
Black	1.280 (16.5)
Schooling n (%)	
Postgraduate studies	2.947 (38.0)
College degree	1.358 (17.6)
High school	3.433 (44.4)
Income in minimum wages *per capita* n (%)	
≤ 3 Wages	3.818 (49.3)
> 3 Wages	3.920 (50.7)
Marital status n (%)	
With a partner	5.307 (68.6)
Without a partner	2.341 (31.4)
Stress at work n (%)	
Low-strain job	1.718 (22.2)
Active job	1.367(17.7)
Passive job	3.031 (39.2)
High-strain job	1.622 (20.9)
Social support in the workplace n (%)	
High	3.427 (44.3)
Low	4.311 (55.7)
Weekly working hours n (%)	
≤ 40 hours	5.060 (65.4)
> 40 hours	2.678 (34.6)
Nature of occupation n (%)	
Manual	1.251 (16.2)
Routine non-Manual	2.213 (28.6)
Non-Manual, non-routine	4.274 (55.2)
Time-related work-to-family conflict n (%)	
Never	3.029 (39.1)
Sometimes	2.375 (30.7)
Often	2.334 (30.2)
Strain-related work-to-family conflict n (%)	
Never	3.741 (48.3)
Sometimes	2.365 (30.6)
Often	1.632 (21.1)
Family-to-work conflict n (%)	
Never	5.168 (66.8)
Sometimes	2.049 (26.5)
Often	521 (6.7)
Lack of time for self-care and leisure n (%)	
Never	2.817 (36.4)
Sometimes	2.550 (33.0)
Often	2.371 (30.6)
Body mass index n (%)	
Eutrophic	3.003 (38.8)
Overweight	3.074 (39.7)
Obesity	1.661 (21.5)
Lifestyle indicator n (%)	
Healthy	5.427 (70.1)
Less healthy	2.311 (29.9)
Presence of comorbidities n (%)	
No	2.521 (32.6)
Yes	5.217 (67.4)

SD: standard deviation.

 The behavior of SRH over time was stable, with the majority of transitions occurring between the immediately preceding or following categories. Transitions in category 3 (fair) were more balanced between permanence or transition to category 2 (good). There was also a very low frequency of occurrences for categories 4 (poor) and 5 (very poor) at all the times observed ( Figure 2 ). 

Note: The number of participants with very good. good. fair. poor and very poor self-rated health at each time point was respectively: beginning - 2.356. 4.131. 1.144. 89. 18; midpoint - 2.380. 4.207. 887. 63. 15; and endpoint - 2.262. 4.084. 847. 52. 5.

**Figure 2.** Distribution and transitions of self-rated health at baseline (point 1). midpoint (point 6) and endpoint (point 11) of the follow-up period. Brazilian Longitudinal Study of Adult Health (ELSA-Brasil. 2008–2020).

 After applying LCGM, the participants were classified into three patterns of relatively stable SRH evolution over the period evaluated, with the highest means representing the worst self-rated health ( Figure 3 ). Pattern 1 (n = 2,249; 29%), called “good,” included participants who were consistently positive about their health over time (Mean = 1.42; SD = 0.57 points). Pattern 2 (n = 4,715; 61%), called “fair,” had a higher frequency, comprising people who rated their health less positively than those in pattern 1 (Mean = 1.96; SD = 0.61 points). Pattern 3 (n = 774; 10%), called “poor,” comprised people who rated their SRH less positively than patterns 1 and 2, respectively (Mean = 2.62; SD = 0.74 points). Within each pattern, the respondents showed some variability in their SRH, with a slight worsening trend in patterns 1 and 2 over the time assessed ( Figure 3 ). 

**Figure 3.** Mean self-rated health (SRH) for each trajectory pattern identified in the 11 years of follow-up. Brazilian Longitudinal Study of Adult Health (ELSA-Brasil, 2008–2020; n = 7,738)

Regarding the fair SRH pattern, the multinomial logistic regression models, adjusted for all the other variables, showed that higher odds were observed among those with lower income and those with high school or college education, compared to those with postgraduate degrees. Regarding job aspects, high strain job and passive job (compared to low-strain job), as well as low social support increased the odds of belonging to the fair SRH pattern, while working > 40 hours/week proved to be protective. In addition, participants with a less healthy lifestyle, who were overweight or obese and had comorbidities were more likely to belong to the fair SRH pattern, compared to the good trajectory pattern (Table 2).

For the poor SRH trajectory pattern, the adjusted regression models showed higher odds (around 30%) for women, those who declared themselves Brown/Mixed-race, with lower income, who reported low social support in the workplace, frequent work-to-family conflict related to strain, and occasional family-to-work conflict. Higher odds (50%) of belonging to the poor SRH pattern were observed among those who had studied up to high school, those classified as working passively, who reported frequent family-to-work conflict, and those classified as having a less healthy lifestyle. In addition, even higher odds (around 75%) of belonging to the poor SRH trajectory pattern were observed for those classified as having high job strain and being overweight. Odds of around 80% of a poor SRH pattern were also observed for those who frequently reported a lack of time for self-care and leisure. Manual workers and those with comorbidities were twice as likely to belong to the poor pattern. The presence of obesity increased the chance of belonging to the poor SRH pattern fourfold. Yesilarly to what was observed in the regular trajectory, working hours > 40 hours/week also proved to be protective (Table 2).

**Table 2.** Odds ratios and respective 95% confidence intervals of multinomial logistic regression models in the association between sociodemographic, behavioral, and occupational factors and patterns of self-rated health trajectories. Brazilian Longitudinal Study of Adult Health (ELSA-Brasil, 2008–2020).

Baseline variables	**Fair trajectory pattern**	**Fair trajectory pattern**	**Poor trajectory pattern**	**Poor trajectory pattern**
	Crude model OR (95%CI)	Adjusted model OR (95%CI)	Crude model OR (95%CI)	Adjusted model OR (95%CI)
Age	1,00 (0,99–1,01)	1,00 (0,99–1,01)	**1,01 (1,00–1,03)**	**1,01 (1,00–1,03)**
Sex				
Male	**1**	1	**1**	1
Female	0,94 (0,85–1,04)	1,01 (0,90–1,13)	**1,28 (1,08–1,51)**	**1,31 (1,08–1,60)**
Self-declared race/color				
White	1	1	1	1
Brown/Mixed-race	**1,29 (1,15–1,45)**	1,02 (0,90–1,16)	**1,94 (1,61–2,35)**	**1,32 (1,08–1,62)**
Black	**1,41 (1,22–1,64)**	0,92 (0,79–1,09)	**2,53 (2,03–3,16)**	1,20 (0,94–1,54)
Education				
Postgraduate studies	1	1	1	1
College	**1,58 (1,37–1,83)**	**1,33 (1,13–1,55)**	**1,47 (1,12–1,91)**	1,05 (0,78–1,40)
Up to high school	**2,09 (1,87–2,34)**	**1,25 (1,04–1,50)**	**3,55 (2,94–4,29)**	**1,53 (1,13–2,08)**
Minimum wage *per capita*				
> 3 minimum wages	1	1	1	1
≤ 3 minimum wages	**1,81 (1,63–2,00)**	**1,16 (1,02–1,33)**	**3,00 (2,53–3,56)**	**1,37 (1,09–1,72)**
Marital status				
Without a partner (a)	1	1	1	1
With a partner(a)	1,10 (0,98–1,22)	1,07 (0,95–1,20)	**0,83 (0,70–0,99)**	**0,86 (0,72–1,05)**
Stress at work				
Low desgaste	1	1	1	1
Active job	0,91 (0,78–1,06)	0,87 (0,74–1,03)	1,30 (0,98–1,74)	1,06 (0,78–1,45)
Passive job	**1,59 (1,39–1,81)**	**1,19 (1,03–1,38)**	**2,56 (2,01;3,26)**	**1,49 (1,15–1,94)**
High desgaste	**1,81 (1,55–2,12)**	**1,32 (1,11–1,57)**	**3,47 (2,66–4,53)**	**1,75 (1,31–2,35)**
Social support in the workplace				
High	1	1	1	1
Low	**1,12 (1,02–1,24)**	**1,18 (1,06–1,31)**	**1,33 (1,12–1,57)**	**1,36 (1,13–1,63)**
Working hours				
≤ 40 hours/week	1	1	1	1
> 40 hours/week	**0,74 (0,67–0,82)**	**0,85 (0,75–0,96)**	**0,64 (0,53–0,76)**	**0,74 (0,61–0,91)**
**Nature of occupation**				
No manual, no rotineiro	1	1	1	1
Routine non-Manual work	**1,58 (1,41–1,78)**	**1,20 (1,03–1,39)**	**2,23 (1,84–2,70)**	1,21 (0,94–1,55)
Manual labor	**2,43 (2,06–2,86)**	**1,63 (1,32–2,02)**	**4,20 (3,34–5,29)**	**2,08 (1,53–2,84)**
**Time-related work-to-family conflict**				
Never	1	1	1	1
Sometimes	1,00 (0,88–1,12)	-	0,98 (0,80–1,20)	**-**
Often	**0,85 (0,76–0,96)**	-	1,02 (0,84–1,25)	**-**
**Strain-related work-to-family conflict**				
Never	1	1	1	1
Sometimes	0,95 (0,84–1,06)	1,02 (0,89–1,16)	1,05 (0,87–1,28)	1,10 (0,89–1,37)
Often	1,07 (0,94–1,22)	1,16 (0,99–1,37)	**1,49 (1,21–1,83)**	**1,39 (1,08–1,80)**
**Family-to-work conflict**				
Never	1	1	1	1
Sometimes	1,11 (0,99–1,25)	1,12 (0,99–1,27)	**1,31 (1,08–2,57)**	**1,31 (1,07–1,61)**
Often	**1,35 (1,08–1,68)**	1,22 (0,97–1,55)	**2,09 (1,54–2,83)**	**1,58 (1,13–2,22)**
**Lack of time for self-care and leisure**				
Never	1	1	1	1
Sometimes	1,06 (0,94–1,20)	**1,18 (1,03–1,35)**	1,12 (0,91–1,37)	1,25 (0,99–1,58)
Often	1,07 (0,95–1,22)	**1,27 (1,08–1,49)**	**1,53 (1,25–1,87)**	**1,82 (1,41–2,34)**
**Body Mass Index**				
Eutrophic	1	1	1	1
Overweight	**1,65 (1,48–1,85)**	**1,50 (1,34–1,69)**	**2,02 (1,65–2,48)**	**1,73 (1,40–2,13)**
Obesity	**2,81 (2,41–3,27)**	**2,29 (1,95–2,69)**	**6,25 (5,00–7,82)**	**4,25 (3,35–5,39)**
**Lifestyle indicator**				
Healthy	1	1	1	1
Less healthy	**1,58 (1,41–1,78)**	**1,36 (1,21–1,54)**	**2,01 (1,68–2,39)**	**1,57 (1,30–1,89)**
**Presence of comorbidity**				
No	1	1	1	1
Yes	**1,98 (1,76–2,22)**	**1,63 (1,43–1,85)**	**3,28 (2,76–3,90)**	**2,26 (1,86–2,75)**

OR: odds ratio

95%CI: 95% confidence interval

Note: Those with significant associations at the 5% significance level appear in bold.

Reference trajectory pattern = good. The multiple model was adjusted for all the variables. and those with statistical significance and a significant contribution to the model were kept in the final model. The variable “work-family conflict (time)” was not kept in the final adjusted model. as its removal contributed to a better model fit.

## DISCUSSION

 The three patterns of SRH trajectories that emerged through the application of the LCGM were relatively stable, with a slight worsening trend in the patterns of poor and good SRH trajectories over time. This result is consistent with other international studies on health trajectories, which have also shown that most people have stable trajectories over time, but that there are smaller groups with declining and improving trajectories ^4^^,^^2020^^,^^2012^ . 

In this study, around 10% of the interviewees followed a consistently poor SRH trajectory over the time assessed. When assessing the characteristics associated with a poor trajectory, female gender, worse socioeconomic and occupational conditions, less healthy lifestyle, and the presence of comorbidities stood out. Similar characteristics, but with attenuated odds, were observed in the fair SRH trajectory pattern.

 Regarding sociodemographic variables, female gender, Brown/Mixed-race, lower schooling, and lower income were associated with the worst patterns of SRH trajectories. The association between the schooling and income variables and patterns of trajectories shows how inequalities can affect the population’s health. In this perspective, the National Household Sample Survey (2016) ^2022^ showed that the Black and Mixed-race populations have less schooling and, when employed, generally receive half the income compared to the White population ^2017^ . It is important to note that in the crude regression analyses, Black people also had higher odds of poor and fair SRH. It is likely that adjusting for schooling and/or income contributed to the association between Black people and poor SRH losing statistical significance in the multiple models, since income and schooling can be mediators of the association between race and SRH. 

 The association of worse socioeconomic conditions with worse patterns of SRH trajectories has also been found in other studies ^2022^^,^^8^ . A recent study carried out in the United Kingdom showed that an increase in income has a positive impact on self-rated health trajectories ^2022^ . Socioeconomic conditions influence health in different ways, whether in the acquisition of health goods and services, access to health services, or housing, eating habit, and lifestyle conditions ^2022^^,^^2021^ . In this way, these conditions are closely interlinked and have an impact on health. 

 Although a direct relationship between aging and worse self-rated health is expected, in this study no associations were observed between age and SRH trajectory patterns. It is worth noting that this sample is made up only of active workers throughout the study’s follow-up period, which may have contributed to reducing the effect of age ^2017^ , given that older workers were excluded based on the retirement criterion. 

 Unlike studies that only look at the association of SRH trajectories with socioeconomic or health factors, this study makes an important contribution to the literature by also evaluating the association of SRH with occupational factors. In this approach, passive or high-strain work, low social support, Manual occupation, work-to-family or family-to-work conflict, and lack of time for self-care and leisure were associated with the worst health trajectory patterns. Occupational characteristics related to work stress, type of occupation and social support in the workplace were also associated with worse SRH trajectories in two other studies ^2010^^,^^2006^ . A Brazilian study ^2021^ found that people with active, passive, and high-strain jobs had a lower risk of changing their SRH from fair to good. The study also found that the group with low social support in the workplace was less likely to migrate from poor to good SRH over time. 

 Overall, the findings reinforce the idea that aspects of work can impact on workers’ health in different ways. Low occupational status, high demand, and low control at work are directly related to the occurrence of work-related stress. In addition, low social support in the workplace contributes to enhancing the effects of work-related stress ^2017^ , increasing the risk of physical and mental illness ^2022^ . 

 In addition to aspects of the work environment, conflict between work and family demands was associated with worse patterns of SRH trajectories. Some studies have looked at the association between work-family conflict and poor SRH, with worse associations being found in women ^2010^^,^^2017^ . A study that looked at the relationship between conflict and SRH trajectories showed that those with lower educational levels who reported being exhausted at work were more likely to have health trajectories classified as “poor,” compared to people with higher educational levels ^2014^ . 

 Regarding working hours, our results showed that working more than 40 hours/week was protective against the worst health trajectory pattern. A longitudinal study carried out in Korea showed that long working hours (> 52 hours) are associated with a worsening of SRH over time for both sexes. However, the authors also found that only among women ^2018^ , working less than 40 hours a week was associated with worse health trajectories. There is still no consensus on the association between working hours and SRH. Our hypothesis for the results is that long working hours may be associated with good SRH, since healthier people are able to work longer hours per week. Considering that most people have stable trajectories over time ^4^^,^^2020^^,^^2012^ , it is possible that shorter working hours are a reflection of poor health. Therefore, the associations seem to differ according to the type of study, population, and gender ^2018^^,^^2011^ . 

 The fact that such aspects of health as obesity, a less healthy lifestyle and the presence of comorbidities were also associated with the worst pattern of SRH trajectories is in line with other studies ^4^^,^^2012^ that have observed associations between unhealthy lifestyle habits and inadequate diet and the worst health trajectories. The relationship between obesity and the worst patterns of SRH trajectories can be explained by obesity-related comorbidities, given that obesity is associated with a number of diseases. Additionally, obesity is also related to less healthy health behaviors, which can lead to a poor quality of life and, consequently, worse SRH ^2020^ . Moreover, the tendency for people with obesity to rate their health negatively may be related to the increase in information about the negative consequences for their health ^2016^ . Notably, these behaviors do not all necessarily have the same importance in promoting health (e.g., smoking daily is more harmful to health than not eating fruit daily) and here they have been evaluated as such. However, this indicator is an attempt, albeit simplistic, to analyze behaviors simultaneously, which is closer to what defines lifestyle in people’s reality. 

 This study stands out for identifying patterns in SRH trajectories using the latent class growth model, which is still little used, and for analyzing SRH over 11 time points for the first time in the Brazilian population. It should be noted that this study included the analysis of lifestyle variables, as recommended in a recent study ^6^ . Additionally, the study population comprised active workers from a large Latin American cohort, which offers rare opportunities for investigation, such as the evaluation of occupational characteristics, in addition to the socioeconomic and health factors already explored in the literature. 

As for limitations, the findings should be generalized with caution since the results refer to a cohort of civil servants. The stability of the participants in terms of employment and income may have influenced the low proportion of poor and very poor SRH. Another limitation refers to the exposure variables assessed only at baseline. However, we believe that the high stability of civil servants may have attenuated the effect of variability, especially in sociodemographic characteristics. Finally, although the study used self-reported data, which may be subject to bias, validated instruments and a rigorous quality assurance and control process were used throughout all phases.

## CONCLUSIONS

This article contributes to understanding the factors associated with patterns of self-rated health in active workers, which is still scarce in the literature. The results showed three patterns of trajectories, the most adverse of which were related to worse socioeconomic and occupational conditions, even after adjusting for more proximal variables such as lifestyle, excess weight, and comorbidities. The results reinforce the importance of drawing up public policies aimed at minimizing social inequalities and increasing health promotion in the Brazilian population, factors that are widely acknowledged. In addition, the study innovates by including work-related variables, pointing to the need for policies that promote a healthy working environment, combined with a balance between work demands and personal life, factors with great potential for intervention, especially in the current scenario of increasingly postponing retirement and keeping workers active for longer.
